# Management of systemic sclerosis: British Society for Rheumatology guideline scope

**DOI:** 10.1093/rap/rkad022

**Published:** 2023-03-14

**Authors:** Christopher P Denton, Enrico De Lorenzis, Elen Roblin, Nina Goldman, Begonya Alcacer-Pitarch, Emma Blamont, Maya Buch, Maresa Carulli, Caroline Cotton, Francesco del Galdo, Emma Derrett-Smith, Karen Douglas, Sue Farrington, Kim Fligelstone, Luke Gompels, Bridget Griffiths, Ariane Herrick, Michael Hughes, Clare Pain, Georgina Pantano, John Pauling, Athiveeraramapandian Prabu, Nuala O’Donoghue, Elisabetta Renzoni, Jeremy Royle, Muditha Samaranayaka, Julia Spierings, Aoife Tynan, Louise Warburton, Voon Ong

**Affiliations:** Centre for Rheumatology, Division of Medicine, University College London, London, UK; Department of Rheumatology, Leeds Institute of Rheumatic and Musculoskeletal Medicine, University of Leeds, Leeds, UK; Centre for Rheumatology, Royal Free London NHS Foundation Trust, London, UK; Centre for Rheumatology, Division of Medicine, University College London, London, UK; Department of Rheumatology, Leeds Institute of Rheumatic and Musculoskeletal Medicine, University of Leeds, Leeds, UK; Scleroderma and Raynaud’s UK, London, UK; Department of Rheumatology, University of Manchester, Manchester, UK; Department of Rheumatology, Hammersmith Hospitals NHS Foundation Trust, London, UK; Department of Rheumatology, Liverpool University Hospitals NHS Foundation Trust, Liverpool, UK; Department of Rheumatology, Leeds Institute of Rheumatic and Musculoskeletal Medicine, University of Leeds, Leeds, UK; Department of Rheumatology, University of Birmingham, Birmingham, UK; Department of Rheumatology, Dudley Group NHS Foundation Trust, Birmingham, UK; Department of Rheumatology, University of Manchester, Manchester, UK; Centre for Rheumatology, Royal Free London NHS Foundation Trust, London, UK; Department of Rheumatology, Somerset NHS Foundation Trust, Taunton, UK; Department of Rheumatology, Freeman Hospital, Newcastle, UK; Department of Rheumatology, Hammersmith Hospitals NHS Foundation Trust, London, UK; Department of Rheumatology, Hammersmith Hospitals NHS Foundation Trust, London, UK; Department of Rheumatology, Alder Hey Children’s Hospital, Liverpool, UK; Patient representative, London, UK; Department of Rheumatology, North Bristol NHS Foundation Trust, Bristol, UK; Department of Rheumatology, University of Birmingham, Birmingham, UK; Department of Rheumatology, Salford Royal NHS Foundation Trust, Salford, UK; Interstitial Lung Disease Unit, Royal Brompton NHS Foundation Trust, London, UK; Department of Rheumatology, University Hospitals NHS Foundation Trust, Leicester, UK; Department of Rheumatology, Salford Royal NHS Foundation Trust, Salford, UK; Department of Rheumatology, University of Utrecht, Utrecht, The Netherlands; Centre for Rheumatology, Royal Free London NHS Foundation Trust, London, UK; Primary Care Sciences, Keele University, Keele, UK; Centre for Rheumatology, Division of Medicine, University College London, London, UK

**Keywords:** scleroderma, SSc, pulmonary fibrosis, guideline, management

## Abstract

This guideline will provide a practical roadmap for management of SSc that builds upon the previous treatment guideline to incorporate advances in evidence-based treatment and increased knowledge about assessment, classification and management. General approaches to management as well as treatment of specific complications will be covered, including lung, cardiac, renal and gastrointestinal tract disease, as well as RP, digital vasculopathy, skin manifestations, calcinosis and impact on quality of life. It will include guidance related to emerging approved therapies for interstitial lung disease and account for National Health Service England prescribing policies and national guidance relevant to SSc. The guideline will be developed using the methods and processes outlined in Creating Clinical Guidelines: Our Protocol. This development process to produce guidance, advice and recommendations for practice has National Institute for Health and Care Excellence accreditation.

##  


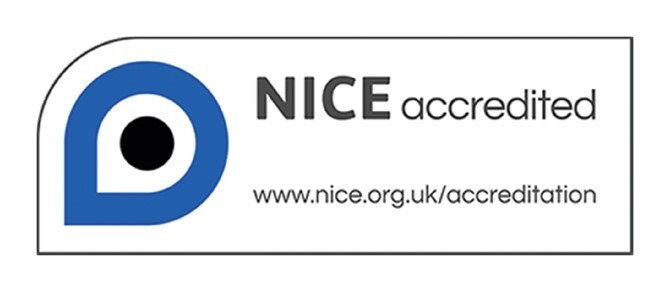


NICE has accredited the process used by BSR to create its clinical guidelines. The term began on 27 February 2012 and the current renewed accreditation is valid until 31 December 2023. More information on accreditation can be viewed at www.nice.org.uk/accreditation.

## Why the guideline is needed

The current British Society for Rheumatology (BSR) guideline for SSc was published in 2016 [1] and represented an important step forward for management of this complex disease with high morbidity, mortality and unmet medical need. It provides a roadmap for best practice management to try and harmonize treatment and investigation of SSc and defines key quality and audit standards that can be used to assess practice and improve outcomes.

Updating the guideline is now required to conform to the National Institute for Health and Care Excellence accreditation process that applies to BSR guidelines and has important implications for application and implementation across National Health Service (NHS) England. The guideline will be developed using the methods and processes outlined in Creating Clinical Guidelines: Our Protocol [2].

Moreover, there have been important new trials and evidence-based therapies (e.g. nintedanib, tocilizumab and soon rituximab for lung fibrosis) as well as changes in NHS England prescribing policies (e.g. digital ulcers) that mean the present guideline no longer reflects current best practice and does not reflect all the available high-quality evidence that can underpin management of SSc.

The previous guideline did not include children and young people. In line with other BSR guidelines and equality considerations, we feel it is important to also represent the needs of children and young people and update the guideline to include all ages of people affected by SSc.

## Key facts and figures

SSc affects ≈1–2 in 10 000 of the UK population and is complex and diverse, with limited treatment options [3]. It has the highest mortality of any of the autoimmune rheumatic diseases, with approximately half of people affected by SSc eventually dying as a direct result of the disease or a related complication [4]. Approximately 1 in 3 people with SSc develops lung fibrosis and 1 in 10 may develop pulmonary hypertension, and these are currently the most frequent direct causes of SSc-related death [5]. Of people with SSc, 1 in 5 develops overlap connective tissue diseases, which require specific management in parallel with SSc. It is plausible that vigilant screening for organ-based complications and routine use of disease-modifying immunosuppression in dcSSc have improved overall survival, and this is supported by single-centre observational cohort analysis [6].

## Current practice

Current management is generally centred on rheumatology clinics in a secondary care setting with appropriate involvement and cross-referral to other specialties including dermatology, respiratory medicine, gastroenterology and others. There are important shared care links with other specialists. Treatment may include centres commissioned for specialized rheumatology and may also involve national designated centres for specific complications such as pulmonary hypertension. There are many management challenges that will be considered in the BSR treatment guideline to harmonize care and define auditable standards.

More systematic approaches to investigation and treatment of major complications such as scleroderma renal crisis or pulmonary arterial hypertension (PAH) likely explain this better outcome. The availability of a series of best practice recommendations that were published by the UK Systemic Sclerosis Study Group, together with evidence-based recommendations from EULAR/EUSTAR that were published in 2009 [7] and updated in 2015 [8], have helped to harmonize management across expert centres. The BSR treatment guideline provides a template for management within the UK NHS since it was published in 2016 [1]. Some available treatments such as autologous haematopoietic stem cell transplantation need very specialist centres and a framework of multidisciplinary and multiprofessional care is required.

Challenges in current practice are early diagnosis, timely referral to secondary care, integrated management with shared care, including appropriate tertiary centres and specialists, and ongoing management of disease burden and non-lethal morbidity.

Recent studies suggest a treatment landscape of supportive or symptomatic treatment in most patients diagnosed with SSc, but also highlight the challenge of delayed diagnosis in those patients with milder or less complete disease that may fulfil proposed criteria for very early diagnosis of SSc [9]. Another challenge is those presenting with a major organ-based complication such as thrombotic microangiopathy, PAH or interstitial lung disease (ILD) but who have not yet been diagnosed with SSc although they may fulfil the 2013 ACR/EULAR classification criteria [10].

For dcSSc skin disease, most people are treated with immunosuppression along the lines explored in the large European Scleroderma Observational Study [11] with MMF, MTX or sometimes intravenous CYC. Immunosuppression is also routinely used for SSc-ILD based on expert opinion and published clinical trial data. Recent clinical trials have confirmed or suggested the benefit of other approaches to SSc-ILD, including nintedanib, tocilizumab and rituximab [12]. These will be reviewed in the scope of the updated BSR guideline.

## Who the guideline is for

This guideline is for: rheumatologists, dermatologists, respiratory physicians and other clinicians involved in management of people with SSc; specialist nurses and allied healthcare professionals involved in caring for people with SSc; people with SSc and primary care clinicians.

### Equality considerations

All ages and ethnicities are affected by scleroderma, but the need for high-quality education, long-term community-based management and the use of specialized treatments and ongoing screening means that language and cultural barriers to equitable and excellent care need to be considered and addressed.

## What the guideline will cover

### Who is the focus?

#### Groups that will be covered

The guideline will cover people of all ages with SSc. Relevance to young people with SSc will also be considered with inclusion of relevant paediatric rheumatology expertise in the working group and dissemination of the guideline to all stakeholders. This will include lcSSc, dcSSc, SSc sine scleroderma and overlap SSc fulfilling classification criteria (EULAR/ACR 2013) [8].

#### Groups that will not be covered

Treatment of localized scleroderma (morphoea) and of ‘scleroderma-like’ conditions (e.g. scleroedema, scleromyxedema, fasciitis, nephrogenic systemic fibrosis) will not be considered in this guideline.

### Settings

#### Settings that will be covered

Settings that will be covered include SSc in hospital-based settings including secondary care rheumatology, other specialized care including tertiary hospital settings and in communities of shared care and primary care settings.

### Key areas that will be covered

We will look at evidence in the following areas when developing the guideline, but it may not be possible to make recommendations in all the areas: early diagnosis, classification and stratification of risk; global management of SSc; treatment of organ-based complications of SSc, both drug and non-drug; and organizations or services for SSc within the NHS, including paediatric services and transition of paediatric patients to adult services.

### Related guidance

Related guidance includes the published BSR guideline (2016) [1], EULAR recommendations (published 2009; updated 2016) [6, 7], European Dermatology Forum Guideline (Part 1, 2017) [13], UKSSG best practice recommendations (gastrointestinal complications, cardiac disease, renal crisis, digital vasculopathy) [14–17] and the Single Hub and Access point for paediatric Rheumatology in Europe recommendations on juvenile SSc [18].

### Key issues and draft questions

The working group has identified the following key issues and draft questions related to them. The key issues and draft questions will be used to develop more detailed review questions, which will guide the systematic review of the literature.

## Proposed updated guideline structure

### General approach to SSc management

The working group recognizes the importance of timely diagnosis of SSc and that there have been advances in understanding early identification of the disease. Delays in diagnosis should be minimized. As previously, it is appropriate that early dcSSc is a particularly important diagnosis because of the early risk of severe internal organ complications and the need for specialist referral and initiation of disease-modifying treatment with immunosuppression.

### Current priorities and approach

- What is the best approach to timely diagnosis and specialist referral?
- How can people with SSc be classified for stratified medicine and management?
- What are the best treatments for early dcSSc?
- When and how should people with SSc be screened for concurrent malignant disease?
- What non-pharmacological treatments are supported by evidence, including vitamins, supplements, massage (manual lymph drainage) and psychological support for early new diagnosis and established disease?

### Key therapies and treatment of organ-based disease in SSc

This section will update and expand the management and treatment recommendations included in the previous BSR guidelines. Trials for several new treatments have been published for complications such as SSc-ILD and PAH. In addition, new approaches to treatment with established therapies are likely to be considered, including autologous haematopoietic stem cell transplantation. These topics will be considered in the following questions:

#### Autologous stem cell transplantation

- Which people with SSc should be considered for autologous stem cell transplantation, taking into account the risks as well as potential short- and long-term benefits?

#### Cardiopulmonary complications

- How should ILD in SSc be managed and treated, taking into account new approved drugs such as nintedanib with emerging evidence supporting the use of targeted biological agents?
- What is the best evidence-based management and treatment for pulmonary hypertension, including PAH?
- What is the best evidence-based management and treatment for cardiac involvement?

#### Digital vasculopathy

- What is the best evidence-based management and treatment for Raynaud’s phenomenon (RP)?
- What is the best evidence-based management and treatment for digital ulceration?
- What is the best evidence-based management and treatment for critical digital ischaemia?

#### Gastrointestinal tract disease

- What is the best evidence-based management and treatment for gastrointestinal complications, including nutrition, oropharyngeal and dental aspects (sicca syndrome)?

#### Renal complications

- What is the best evidence-based management and treatment for scleroderma renal crisis?

#### Skin complications

- What is the best evidence-based management and treatment for skin manifestations in addition to skin thickening and fibrosis such as telangiectasis and pruritis?
- What is the best evidence-based management and treatment for calcinosis in SSc?

#### Neurological complications

- What is the best way to manage and treat neurological complications of SSc, including peripheral neuropathy, allodynia, cranial neuropathy and neuralgia?

#### Musculoskeletal disease, fatigue and quality of life

- What is the best way to manage and treat musculoskeletal manifestations of SSc, including soft tissue loss, contractures, arthritis and bone health?
- What is the best evidence-based management and treatment for pregnancy and reproductive health problems, including male and female sexual dysfunction?
- What are the best interventions for general impact of SSc on health status and quality of life, including fatigue?

### Service organization and delivery within NHS England and devolved nations

The working group recognizes that there are challenges delivering high-quality equitable care for SSc across England and the devolved nations. This reflects the infrequency of SSc in primary and secondary care and its clinical diversity, as well as the need for comprehensive multispecialist and interdisciplinary clinical care. In addition, while treatment options are increasing, it is recognized that clinical impact can be limited, and even with optimal management there is a significant unmet medical need for people with SSc.

The group will provide feasible and appropriate ways of benchmarking delivery of care and assessing standards so that audits of service delivery as well as patient-specific evaluations can be undertaken in a robust and standardized way. It is likely that SSc will provide a template that may be relevant to other uncommon multisystem autoimmune rheumatic diseases managed across the NHS.

### Approaches to audit of the guideline

#### What are reasonable key quality standards?

How can an audit of the guideline be best performed to assess service delivery and to undertake a patient-specific audit?

The guideline is expected to be published in 2024.

## Guideline working group

Chris Denton (chair), Voon Ong (rheumatologist), Ariane Herrick (rheumatologist), Maresa Carulli (rheumatologist), Francesco del Galdo (rheumatologist and EUSTAR guideline liaison), Elizabeth Renzoni (respiratory physician), John Pauling (rheumatologist), Emma Derrett-Smith (rheumatologist), Jeremy Royle (rheumatologist), Muditha Samaranayaka (rheumatologist), Nuala O’Donoghue (dermatologist), Michael Hughes (rheumatologist), Athiveeraramapandian Prabu (rheumatologist), Karen Douglas (rheumatologist), Bridget Griffiths (rheumatologist), Maya Buch (rheumatologist), Clare Pain (paediatric rheumatologist), Julia Spierings (autoimmune disease stem cell transplant expert), Luke Gompels (rheumatologist), Caroline Cotton (BSR Standards, Audit and Guidelines Working Group liaison), Aoife Tynan (pharmacist), Kim Fligelstone (patient representative), Georgina Pantano (patient representative), Begonya Alcacer-Pitarch (allied healthcare representative), Louise Warburton (general practitioner), Emma Blamont and Sue Farrington (Scleroderma & Raynaud’s UK patient organization), Nina Goldman (respiratory medicine fellow), Enrico De Lorenzi (rheumatology fellow) and Elen Roblin (rheumatology fellow).

## Data Availability

No new data were generated in support of this work.
